# Indian Psychiatry and classification of psychiatric disorders

**DOI:** 10.4103/0019-5545.69221

**Published:** 2010-01

**Authors:** K. S. Jacob

**Affiliations:** Department of Psychiatry, Christian Medical College, Vellore, India

**Keywords:** Classification, Indian psychiatry, Mental disorders

## Abstract

The contribution of Indian psychiatry to classification of mental disorders has been limited and restricted to acute and transient psychosis and to possession disorders. There is a need for leadership in research in order to match diagnosis and management strategies to the Indian context and culture.

## INTRODUCTION

Classification is a process by which complex phenomena are reduced by rearranging into categories based on shared characteristics. Despite opposition from antipsychiatry and the dynamic schools, classification in psychiatry became a movement and revolutionized the discipline. The advantages of classification include communication, control (if etiology is known) and comprehension. Its disadvantages include the presence of mixed states and artificial boundaries leading to a superficial understanding and stigma and misuse through labeling. The many important issues related to classification include those concerning validity, reliability, feasibility and coverage. These abstractions and concepts change with time and need constant revision to keep up with the available evidence. This article attempts to summarize articles on classification which have appeared in the Indian Journal of Psychiatry over the past 50-odd years and also mention other significant studies related to classification which have been published from India.

## MATERIALS AND METHODS

The website of the Indian Journal of Psychiatry (http://www.indianjpsychiatry.org/search.asp), which has all the past issues of the journal online, was searched using a search strategy with the key word “classification”. The search retrieved five with the word in the title, five with the word in the abstract and 81 with the word in the text. Of the articles with the word classification in the text, only five had a 100% match. All articles were reviewed for relevance. Other relevant publications have also been examined and cited.

## RESULTS

Indian work has influenced international classifications of mental disorders. The two areas where significant contributions have been made to classification of mental disorders include the introduction of the category acute and transient Psychosis and the Dhat syndrome. These are discussed in some detail while the others are briefly mentioned.

### Acute psychosis

Wig and Singh extracted psychiatric categories from the APA DSM II relevant for use in India.[1] They also argued for the category of acute psychosis for brief episodes precipitated by stress, which does not fit into the Kraepelinian dichotomy. They cited Asian, German and Scandinavian work in support of a clinically different group of psychosis whose presentations and outcome differed from that of schizophrenia and manic depression. They sub-classified acute psychosis into confusional, paranoid hallucinatory, schizoaffective and also mentioned hysterical psychosis. The category of acute Psychosis was also reiterated by Teja.[2] He also highlighted the need for a category of acute psychosis of psychogenic and uncertain etiology. He subcategorized it into reactive depressive psychosis, reactive excitation, acute paranoid reaction and reactive confusion.

Psychosis of brief duration are commonly seen in the developing world and pose a challenge to clinicians. Such atypical psychosis have been historically described in literature under a variety of diagnostic labels. Many studies on acute and transient psychosis have been done in India and have been extensively reviewed by Malhotra.[3] The first major study which recognized the problem of acute onset of psychosis with a good prognosis was the International Pilot Study of Schizophrenia (IPSS) (1968-70).[4] The Agra center contributed to this international multi-center investigation. The main finding in relation to acute and transient psychosis was the fact that the course and outcome of people living in the developing world was better than those living in developed countries. About a quarter of people diagnosed to have schizophrenia had only one episode and good outcome. The findings of the IPSS raised the question as to whether these subjects with good outcome had a separate psychosis or were they part of the schizophrenia group.

The Determinants of Outcome of Severe Mental Health Disorders (DOSMeD) (1978-80), although designed to study first onset psychosis and provide information on the incidence of schizophrenia, also provided findings related to acute and transient psychosis.[5] Chandigarh was the Indian center and part of the multinational effort. The incidence of broadly defined schizophrenia (which included the ICD 9 reactive and unspecified psychosis) was 1.5-4.2/lakh/year compared to narrowly defined schizophrenia (0.7/lakh/year). The incidence of broadly defined schizophrenia which included non-affective, acute and remitting psychosis was 10 times higher in the developing world than in the developed countries. These patients also exhibited a benign course at two-year follow-up.

The cross-cultural study of acute psychosis (CAP) (1980-1982)[6] was an off shoot of the DOSMeD study. The study aimed to differentiate acute and transient psychosis from schizophrenia and manic depressive psychosis. It also aimed to understand its relationship with psychological and physical stress. Its main findings included the fact that 41.2% of patients had symptoms of schizophrenia while affective symptoms were documented in 20% of the sample of 1004 patients with acute psychosis. About 41.7% reported stress at onset and two-thirds of the subjects remained without relapse at one year follow-up. The outcome of patients with schizophrenia symptoms was similar to those with affective presentations.

The Indian Council of Medical Research’s multicenter study of acute psychosis in Bikaner, Goa, Patiala and Vellore documented 52% of patients with acute psychotic presentations who could not be classified as schizophrenia or MDP.[7] The findings of the Chandigarh Acute Psychosis Study were similar with 40% receiving the label of acute psychosis.[8]

These studies provided evidence of a non-affective, non schizophrenia psychosis with remission and good outcome and lead to the inclusion of acute and transient Psychosis as a separate category in the International Classification of Disease 10.[9] Acuteness of onset, brief duration and polymorphic picture were accepted as defining criteria. The presence of stress was coded as an additional feature. Organic conditions, substance abuse and affective disorders were to be excluded.

Studies which have examined the association between stress and vulnerability in acute and transient psychosis have documented that those with higher stress have a lower genetic vulnerability and vice versa supporting the stress vulnerability hypothesis for the development of such psychosis.[10]

The follow-up studies of acute psychosis from Chandigarh have shown that 14 out of 17 patients maintained full recovery throughout the 12 year follow-up.[11] The average duration of acute psychosis was two to four months, which was longer than the one to three months suggested by ICD 10.[12] The recurrence in the DOSMeD cohort of acute and transient psychosis was 11.76% at 12-year follow-up.[11]

Other workers have also followed up patients with acute and transient psychosis.[13,14] A large proportion of patients were later diagnosed to have affective disorder (9.2%), schizophrenia (26.4%) or recurrent episodes of acute psychosis (11.5%), and others did not present with psychotic symptoms over the follow up period suggesting that it is clearly difficult to predict their response to medication, course and outcome. However, it is well known that acuteness of onset is a good prognostic factor in both schizophrenia and mood disorders. They have argued that the concept of acute psychosis is necessary since many patients may present within a short time of the onset of their illness, at which point the clinical features may not allow them to be categorized into any of the more classical disorders. Although many patients recover, some have relapses with similar acute psychotic presentations, a significant proportion also develop schizophrenia and mood disorders. The difficulty in reaching a diagnosis at the time of the initial presentation is because it is often difficult to recognize the classic syndromes at the onset of the illness. However, these can be identified over time as they develop into the syndrome later. Thus, acute Psychosis can be a presentation of the more traditional syndromes. They can also be separate clinical entities, which may or may not recur over time. Assuming that those who present with acute psychosis conform to a homogenous group does not fit in with clinical reality. Work from India has contributed to the introduction of the acute and transient psychosis categories in the ICD 10.

### Dhat syndrome

Wig coined the term “Dhat syndrome” to identify male patients who presented with fatigue, weakness, and multiple somatic complaints.[15] They attributed their symptoms to the loss of semen through nocturnal emission and masturbation. Sexual dysfunction may be associated with the syndrome. The concept, epidemiology, clinical profile, knowledge, attitude, management and work done in India on the syndrome have been reviewed by Prakash.[16]

The syndrome is prevalent in the Indian subcontinent.[17] It was recorded in Europe and USA in the 19^th^ century but has disappeared since.[18] Wig suggested empathic listening, a non confrontational approach, education about misconceptions, support and reassurance in addition to the use of medication for anxiety and depression when required.[15] The syndrome is described in DSM IV Appendix as a culture bound syndrome[19] and under “other specific neurotic disorders” in the ICD 10.

Concepts of illness vary between social groups and different cultures express their symptoms differently. What is regarded as abnormal in western culture may be considered culturally acceptable in non-western societies and vice versa. For example, brief episodes of trance and possession occurring within a religious or culturally accepted situation are normal in the South Asian culture. Cross-cultural variations in presentation of the many syndromes are documented. For example, patients with the *Dhat* syndrome present with a variety of “neurotic” symptoms.[18] These patients also offer “loss of semen” as the explanation for these disabling symptoms. Such patients are diagnosed as *Dhat* syndrome if the physician is aware of the label and the explanation and if he/she focuses on the content.[20] These patients could also receive a label of anxiety, depression, somatization or neurasthenia if the physician emphasizes the form of the presentation. The patient perspective of “loss of semen ” as cause of the symptoms is the explanatory model of his illness.

The culture in South Asia tends to highlight sexual beliefs as cause for a variety of neurotic phenomenon. These explanations generate more acceptance and understanding for the patient than if he highlighted symptoms of anxiety, depression or somatic symptoms per se. Such beliefs are reinforced by traditional Indian systems of medicine which subscribe to these concepts and whose physicians and healers are often the first contact in the “pathway to care ”.

Sexual misconceptions related to *Dhat* are also seen in patients with schizophrenia, substance dependence, bipolar disorders, delusional disorders and major depression. The focus on form allows the psychiatrists to differentiate the different syndromes.[21] International classifications have emphasized form over content as the response to the various treatment modalities is based on the recognition and treatment of the clinical syndrome. This does not imply reduced importance being placed on the person’s culture and beliefs. It mandates the management of the patient’s explanatory model. This is also true for other culture-bound syndromes like *Koro*. Clinicians focusing on content make such presentations appear exotic. Physicians who emphasize on form are able to recognize behavioral syndromes across cultures.

### Psychiatric disorders in primary care

Some authors have highlighted the difficulties in separating anxiety and depression in primary care and general medical facilities.[22,23] The differences in the reality of primary care and the issues include:

1. Differences between patients attending a psychiatric hospital and those who present to primary care. Patients who visit psychiatric facilities often have severe, complex and chronic illness are highly motivated to receive specialist treatment. On the other hand those who visit GPs have milder and less distinct forms of illness with concomitant psychosocial stress.
2. Differing conceptual models and perceptions are employed in different settings. Psychiatrists employ medical models while GPs focus on focus on the psycho-social context, stress, personality and coping.
3. Symptom scores in patients attending primary care, on standardized interview schedules (E.g. Revised Clinical Interview Schedules (CISR) are continuously distributed with no point of rarity between cases and non cases making dichotomous clinical decision making difficult.
4. Mixed presentations of anxiety and depression are common in primary care.
5. Many patients who cross the case threshold do not have the full syndrome attributes of depression or of anxiety.
6. The labeling of patients with sub-syndromal presentations based on distress and impairment essentially implies a lowering of the threshold for diagnosis.
7. Studies using statistical techniques have failed to show superiority of the two-factor anxiety-depression models over the one factor solution. In addition, the anxiety and depression factors of the two-factor model have always been highly correlated.[24]
8. The commonest presentation of psychiatric problems in primary care is with medically unexplained somatic symptoms. However, a significant number of such patients also mention the presence simultaneous psychological stress or distress.
9. The etiology of medically unexplained somatic symptoms is unclear. The general tendency is to assume psychogenesis. However, the label “somatization ” actually acknowledges to medical ignorance rather than understanding.
10. The numerous categories of depression in the International Classification of Disease 10 (ICD 10) for use in psychiatric settings have been clubbed into a single category of depression in the ICD 10 for primary care[25] resulting in patients with features of biological depression are clubbed with normal people with adjustment reactions due to stress and with those who cannot cope with the demands of life because of poor coping skills.
11. Many studies have shown a high rate of spontaneous remission of depression and common mental disorders in primary care. Literature on major depression also supports the argument that there is a high rate of spontaneous remission.
12. Many authors have highlighted the high rate of improvement in the placebo arms of randomized trials employed to test the efficacy of antidepressant medication.
13. Despite efforts at simplification, the guidelines for managing CMD in primary care have proposed elaborate and separate protocols for each of the traditional psychiatric categories making them impractical for routine use.

They have suggested strategies based on the arguments that it is difficult to sub-categorise clinical presentations of common mental disorders in primary care and that current psychiatric treatments are essentially symptomatic and delivered across diagnostic categories.[22,23,26] They support the contention that the presentations currently labeled anxiety, depression or common mental disorders in primary care are illness experiences which do not require disease labels. They make a case for the provision of support without medicalizing the issues. They also suggest that the standards for medical practice should be based on the issues as seen in primary care rather than those employed in tertiary and specialist settings.

The focus on clinical presentations without diagnosis and the symptomatic management of people with emotional distress who present to primary care are complimentary.[22,23,26] These approaches are not new and describe the current practice among competent physicians in primary care. Recent concepts and interventions, based on specialist perspectives, have not only complicated the issues but have disempowered general practitioners with psychiatric jargon and techniques, which are impractical and counterproductive in primary care settings. The reality of primary care, its problems and opportunities demand unique solutions. Transplanting knowledge structure, formations and practices developed and employed in tertiary care and specialist facilities results in a lack of goodness of fit. Context and local knowledge are critical to understanding illness in primary care. Universal abstractions may not fit local reality and artificially force structures. Primary care should be able choose a different framework for the management of psychiatric and emotional problems. Contexts can not only change medical practice but contexts should be able to change medical perspectives.

The complexity of the issues related to the diagnosis and management of such presentations care demand a re-evaluation of the issues. The alternative approaches have to be rooted in primary care in order that they are useful and can be successfully employed.

### Other conditions

The 1970s had a few papers on classification. They reviewed literature, highlighted the prevalent problems of the day and occasionally suggested changes. Many of these concerns are no longer issues with the many changes in international psychiatry over the past four decades. These are briefly mentioned.

Varma highlighted the use of ICD 8 but mentioned the need for some changes for its application in India based on his clinical experience.[27] He highlighted the need to sub-classify hysteria into hysterical conversion and dissociation as was followed in DSM II. He argued for Dhat syndrome as a separate neurotic category and the removal of Ganser syndrome from the hysteria. He also mentioned the need to separate depressive neurosis from depressive reaction.

Rao in his paper reviewed the literature on the classification of depression and discussed classifications by Lewis (1929), Kiloh and Garside (1962), Pollitt (1965) and Kendal (1968) among others.[28]

Singh and Tiwari classified grief into normal (with typical symptomatology and less than six months duration), morbid or pathological (with exaggeration of typical symptoms and duration greater than six months) and complicated grief (with typical neurotic or psychotic illness).[29] They sub-divided morbid grief into chronic, delayed, inhibited, excessive anxiety, excessive guilt, excessive anger, excessive religiosity, identification with the deceased, over idealization and anniversary reaction. Complicated grief was sub-divided into those with hysterical, phobic, obsessive compulsive, manic and acute psychotic episode. These divisions were based on the predominant clinical presentations.

Rao *et al*. described a series of cases and attempted to classify self-injurious behavior (SIB).[30] They argue that SIB is not a single clinical entity and it occurs in various psychiatric syndromes with wide range of psychopathology. They proposed a classification based on clinical criteria related to severity and form. They suggest that the severity of injury seems to be determined by severity of psychopathology and that understanding some of these clinicopsychopathological issues helps in management. They do not propose their classification to be an alternative to existing psychiatric syndromes and categories but as a classification for the diverse SIB seen in clinical practice.

Case reports, case series and epidemiological investigations of possession disorder have been published from India.[31–36] Possession disorders, unique to the developing world, find a place in international classifications as Trance and possession disorder in ICD 10^9^ and as Dissociative disorder NOS.[19]

Occasional papers have also discussed controversial issues related to diagnosis and classification. Issues related to psychiatric co-morbidity and dual diagnosis have been highlighted.[37] Recently debated western categories (e.g. Internet Addiction Disorder) have also been mentioned.[38] A paper by Sarkar *et al*. debates the need to classify suicidal attempts based on the intention to die.[39] Yet, much of the discussion is a repetition the debate in international literature and psychiatry in India is waiting for the final answers from the western world.

## DISCUSSION

Indian psychiatry has accepted the World Health Organization’s ICD-10 classificatory system for routine clinical use. Academic psychiatry in India has been routinely employing the DSM criteria for research. In fact these standards dominate Indian psychiatry. The lists of categories of disorders deliberately converged their codes in recent revisions to make the manuals broadly comparable, though significant differences remain.

Nevertheless, the complex reality of mental illness reduces the impact of the classificatory system employed in clinical practice. The inherently reductionistic nature of all classifications, the heterogeneity within diagnostic categories, the symptomatic nature of all available treatments and the variation in response and outcomes demands individualization of care. From a practical point of view, despite its western origins, the ICD 10 and its predecessors have been employed across cultures with reasonable success. Their nets have been wide and varied and have included diverse concepts and categories allowing for an eclectic approach, which can be tailored to the local cultural context. Many psychiatrists working in the non-western world adopt western medicine using a wide variety of abstractions to suit the local environment.

Western medical and psychiatric concepts, labels and treatments were/are routinely employed in the country. More recently, many Indian psychiatrists and psychiatric centers were involved in the development and field trials of the ICD 10. Consequently, it was assimilated without any difficulty and is used routinely in clinical psychiatric practice. In fact, a few “Indian ” labels were also included in the classification (E.g. Dhat syndrome, Acute and transient psychosis, possession states). The Indian academia has adopted the psychiatric diagnostic and classificatory systems unconditionally. International diagnosis, classification and treatments are the norm. The pressure to keep international scientific advances has resulted in such unconditional acceptance.

Nevertheless, there is a need to examine many of the categories for local relevance. Indian psychiatry needs to focus on its context and culture in order to develop appropriate diagnostic and classificatory systems. This review documents the limited work done in India on the classification of psychiatric disorders. Indian psychiatry should take the lead to study and examine different options rather than wait for western and international answers to be imposed and then willing accept them for want of indigenous solutions.
